# New Insights and Advances in Pathogenesis and Treatment of Very Early Onset Inflammatory Bowel Disease

**DOI:** 10.3389/fped.2022.714054

**Published:** 2022-03-01

**Authors:** Qi-Qi Li, Hui-Hong Zhang, Shi-Xue Dai

**Affiliations:** ^1^The Second School of Clinical Medicine, Southern Medical University, Guangzhou, China; ^2^Department of Gastroenterology, Guangdong Provincial Geriatrics Institute, National Key Clinical Specialty, Guangdong Provincial People's Hospital, Guangdong Academy of Medical Sciences, Guangzhou, China; ^3^Department of Gastroenterology, Guangdong Provincial People's Hospital, Guangdong Academy of Medical Sciences, South China University of Technology, Guangzhou, China

**Keywords:** microRNA, circular RNA, biologics, immunity, gut microbiota

## Abstract

Very early onset inflammatory bowel disease (VEO-IBD) is characterized by multifactorial chronic recurrent intestinal inflammation. Compared with elderly patients, those with VEO-IBD have a more serious condition, not responsive to conventional treatments, with a poor prognosis. Recent studies found that genetic and immunologic abnormalities are closely related to VEO-IBD. Intestinal immune homeostasis monogenic defects (IIHMDs) are changed through various mechanisms. Recent studies have also revealed that abnormalities in genes and immune molecular mechanisms are closely related to VEO-IBD. IIHMDs change through various mechanisms. Epigenetic factors can mediate the interaction between the environment and genome, and genetic factors and immune molecules may be involved in the pathogenesis of the environment and gut microbiota. These discoveries will provide new directions and ideas for the treatment of VEO-IBD.

## Introduction

Very early onset inflammatory bowel disease (VEO-IBD) refers to a subgroup of pediatric patients diagnosed with IBD before the age of 6 years (1); it includes subclasses of infantIBD and neonatal IBD diagnosed before the age of 2 years and 28 days, respectively. Epidemiological data show that the incidence of VEO-IBD has been increasing rapidly, and one study showed that the incidence had reached 7.2% (2). The increasing incidence of VEO-IBD suggests that it is urgent to understand its pathogenesis. The latest and largest genetic association study collected genome-wide association data for over 75,000 patients and controls and identified 163 susceptibility loci for IBD. Interestingly, twin and family studies of IBD showed that for a child with an affected sibling, the risk increases 26 times for Crohn's disease (CD) and increases 9 times for ulcerative colitis (UC). This suggests that both genetic and environmental factors may affect the pathogenesis of VEO-IBD. However, the pathogenesis of VEO-IBD is still not fully clear. To help solve the existing problems, this narrative review starts from the genetic pathogenesis of VEO-IBD, systematically and comprehensively summarizes the existing pathogenesis and treatment, and provides a potential breakthrough point for the therapeutics of VEO-IBD.

## Genetic Factors

### Gene Abnormalities

Owing to technological progress in genetic testing and DNA sequencing, many genome-wide association studies (GWAS) have been improved, showing new single nucleotide polymorphisms (SNPs) (3). However, the explainable susceptibility loci and genetic risk factors discovered thus far only account for 20–25% of the heritability (genetic risk) (4). Moreover, monogenic mutations have been found mostly in children aged under 6 years, and most conventional polygenic IBD patients are older than 7 years.

Discovered in 2001, nucleotide-binding oligomerization domain containing 2 (NOD2) was the first susceptibility gene for CD. It encodes a protein that acts as an intracellular receptor for bacterial products in monocytes and transduces signals activating NFκB. Polymorphisms in NOD2 are one of the greatest genetic risk factors for Crohn's disease. Three different non-synonomous NOD2 polymorphisms, R702 W, G908R, and L1007fsincC, account for ~80% of all NOD2-associated cases of Crohn's disease and they have been reported to cause a loss of receptor function in response to muramyl dipeptide (MDP) stimulation (5). It has been shown that the perception of NOD protein on bacteria is associated with the induction of autophagy (6). DCs from CD patients with susceptibility variants in the NOD2 gene are deficient in autophagy induction, and the localization of bacteria in autophagolysosomes is reduced (7). Additionally, the genome map of CD patients shows that NOD2 deficiency and mutationsare related to CD in the ileum. Therefore, the interaction between ileal microflora and mucosal immunity is changed by NOD2 mutation, which is a high-risk factor for multiple complications of ileal CD and indicates increased susceptibility to CD.

### miRNA Abnormalities

CD and UC have differences not only in the tissue miRNA spectrum but also in the peripheral blood miRNA spectrum. Recently, several studies have analyzed the differential expression of miRNAs in tissue samples and blood between IBD patients and healthy controls, showing that miRNAs may be regarded as novel biomarkers of these diseases (8). Therefore, the recognition of different miRNA expression profiles may provide a method to determine the course of the disease at an early stage.

Serum miR-146a and miR-146b decrease with IFX treatment and long-term glucocorticoid (GC) treatment (weeks) but not with short-term GC treatment (days) (9). Previous studies have shown that miR-146a and miR-146b are responsive to endotoxin, while increasing miR-146a or miR-146b is dependent on inflammatory stimuli (10). miR-146b was previously described as a monitoring biomarker for IBD, positively correlated with endoscopic disease activity, and more specific than serum c-reactive protein (9). In this study, serum miR-320a was found to decrease in response to both infliximab (IFX) treatment and long-term steroids (weeks) but not to decrease during shorter courses of GC treatment. In the resting colonic mucosa of patients with UC and CD, the levels of miRNA miR-320a were higher than those in controls, which may be caused by the sensitivity of the resting colonic mucosa to environmental factors (11).

Normally, miRNA miR-126 decreases with anti-TNF-α and shows a decreasing trend with GCs. A previous study also showed that miR-126 expression is higher in IBD biopsies than in controls and *in vitro*, and overexpression of miR-126 leads to intestinal mucosal barrier dysfunction (12). After the miRNA differential expression changes are confirmed, miRNA may also become a target of future treatment.

### Circular RNAs

Non-coding RNAs (ncRNAs), circular RNAs (circRNAs) produced by reverse splicing of exons from precursor mRNAs, are ncRNAs that mainly act as elements of regulation. Increasing evidence has shown that cyclic RNAs can regulate gene expression through adsorption of miRNAs or interactions with other molecules at the transcriptional or posttranscriptional level. Furthermore, the evolutionary conservation of cyclic RNAs and their specific loop structure formed by phosphodiesters '5 to 3' leads to their resistance tonucleic acid exonucleases, causing a relatively stable expression in the cytoplasm. These features suggest that cyclic RNAs may be ideal biomarkers.

Several studies have shown that circRNA expression dysregulation plays a role in the progression of some cancers and some specific autoimmune diseases (13). CircRNA-004662 has been found to be a better and possible diagnostic biomarker for CD in terms of IBD pathogenesis and it may be a new candidate gene to differentiate CD from UC (14). It has also been found that circRNA-103765 in peripheral blood mono-nuclear cells (PBMCs) of patients with active IBD is significantly upregulated, and IFX treatment can significantly reverse circRNA-103765 expression. *In vitro* studies have shown that TNF-α induces circRNA-103765 expression and it promotes apoptosis, while silencing circRNA-103765 protects against TNF-α-induced apoptosis of human intestinal epithelial cells (ECs). Thus, blocking circRNA-103765 may be a novel approach for the treatment of IBD patients (15). A study found that the expression of circRNA-102685 was upregulated in the colonic tissue of CD patients compared to healthy controls. Therefore, in CD pathogenesis, circRNA-102685 may regulate the expression of target genes through miR-146 (16). HuR (encoded by the Elavl1 gene) has become the main posttranscriptional regulator of intestinal epithelial homeostasis and it is a widely studied RNA binding protein (RBP) (17). To regulate ATG16L1 translation, HuR regulates autophagy by interacting with circPABPN1 in intestinal epithelial cells. Autophagy is generally considered to benefit cell, tissue, and organ homeostasis and it is involved in intestinal mucosal defense and barrier function (18). It has been shown that transcription of CDKN2B-AS11 into circular RNA with specific functions increases cell proliferation, increases cell adhesion, and decreases apoptosis (19, 20). Some studies have found that in UC patients, CDKN2B-AS1 is significantly downregulated, whereas linear and circular CDKN2B-AS1 affects the proliferation of colonic epithelial cells. A decrease in the expression of CDKN2B-AS1 enhances the formation of the colonic epithelium monolayer barrier by destroying Claudin-2 expression (21). Inspired by these studies, a new direction for the targeting of therapeutic drugs is provided by these circRNAs.

## Immune Dysregulation

The gastrointestinal tract is the largest immune organ in the human body, so it is not surprising that people with immunodeficiency have an increased risk of developing IBD. The progress of high-throughput sequencing technology helps describe single gene abnormalities, which change the intestinal dynamic balance through multiple mechanisms, including dysfunction of the epithelial barrier, abnormalities of T and B lymphocytes, a decrease in neutrophils, a defect in the phagocyte ability to kill bacteria and a lack of intestinal innervation.

### Cytokines and Their Receptors

**Figure 1:**
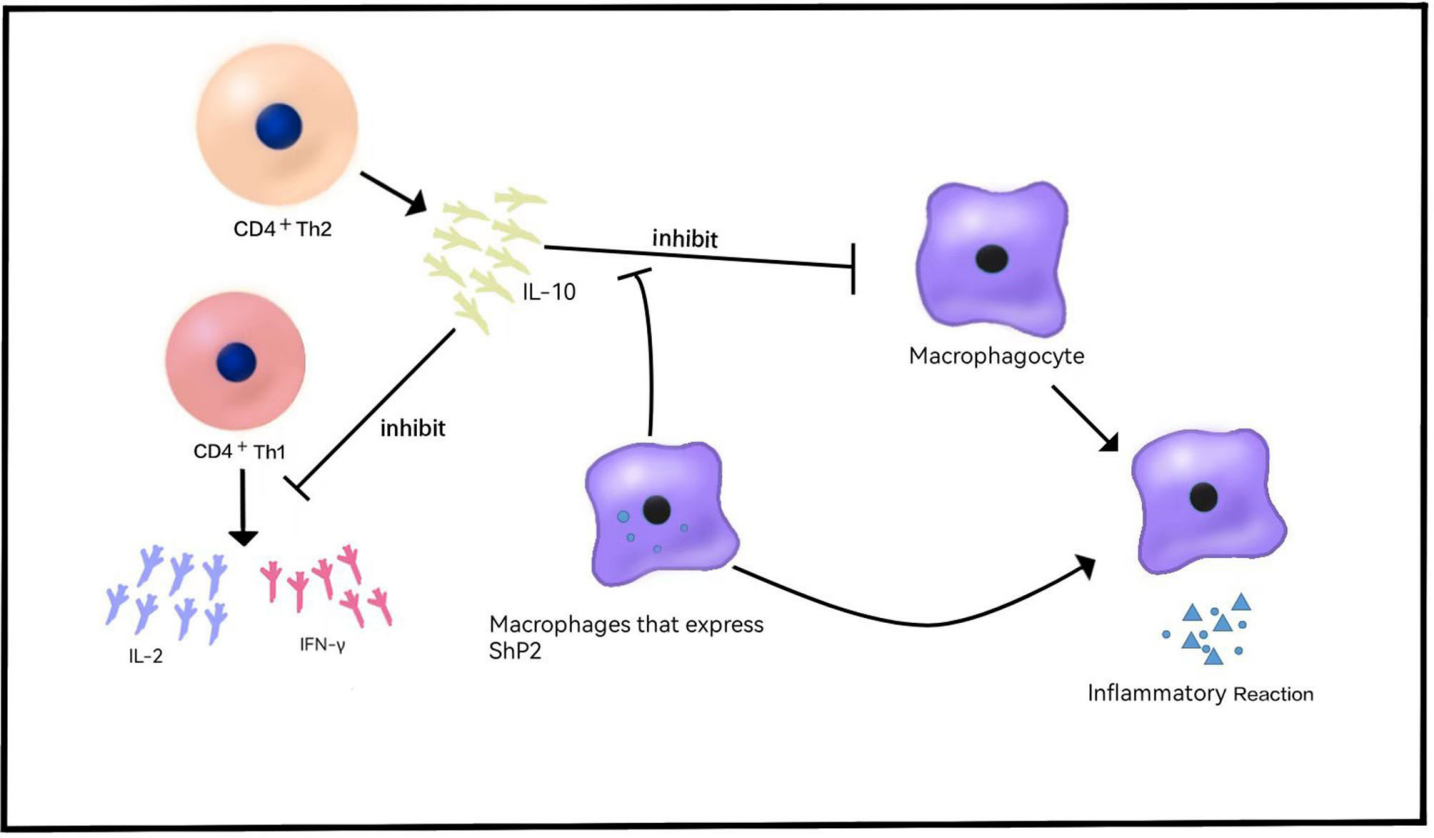
Role of IL-10 in VEO-IBD. IL-10 is released by CD4^+^Th2 cells and inhibits the release of cytokines such as IL-2 and IFN-γ. IL-10 inhibits the release of inflammatory cytokines and the inflammatory response. Shp2 can reduce the sensitivity of macrophages to IL-10 and produce proinflammatory effects.

IL-10 was originally described as a soluble factor released by CD4+ Th2 cells that can preclude the release of CD4+ Th1 cytokines, such as IL-2 and IFN-γ (22) (Figure 1). Subsequently, it was found that IL-10 is secreted by a variety of cells and has multiple effects on T and Blymphocytes, bone marrow cells, etc. In 2009, mutation of IL-10RA-IL-10RB was found in infants with IBD, providing a new comprehension of the pathogenesis of IBD (23), which is consistent with the conclusion of previous studies that the lack of an anti-inflammatory effect of IL-10 causes the activation of an explosive intestinal immune response. These mutations are associated with severe intestinal inflammation, especially in neonatal or infantile VEO-IBD with a phenotype of severe enterocolitis and perianal disease (24). IBD-like immunopathology appears in all patients with IL-10 signaling blocking mutations, showing that these defects are a single gene form of IBD with a penetrance of 100% (24, 25). According to recent immunological studies, IL-10R1 is expressed in many cells involved in innate immunity or acquired immunity, while IL-10RB is an integral part of the receptors of IL-22, IL-26, IL-28 and IL-28β, and it is expressed in immunocytes or non-immunocytes (26). IL-10 polymorphism is associated with the risk of colitis (27), implying that genetic variation in the IL-10-dependent pathway may be related to the pathogenesis of inflammatory bowel disease.

**Figure 2:**
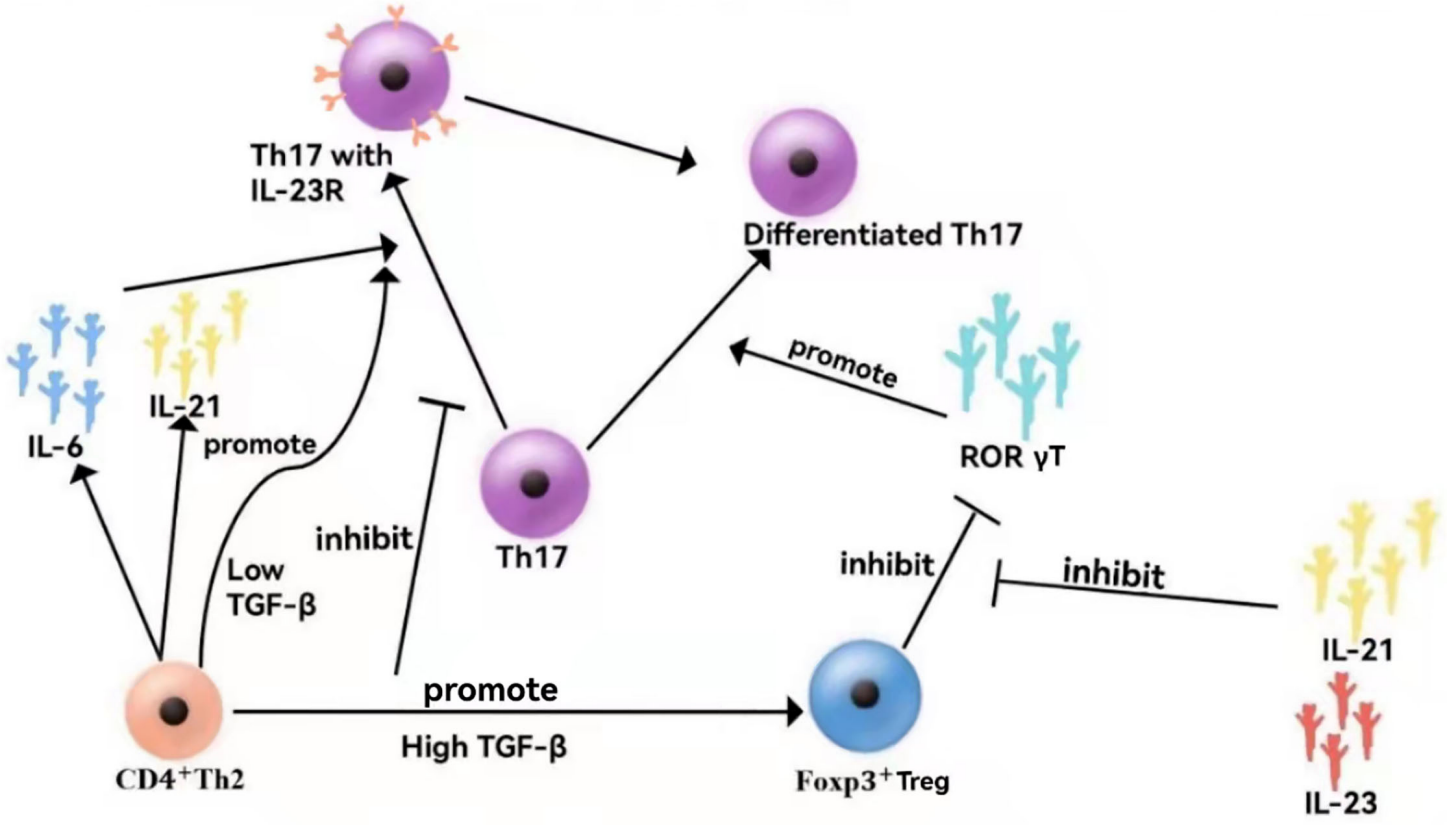
Cytokine networks in VEO-IBD. At low concentrations, TGF synergistically with IL-6 and IL-21 promotes the expression of the IL-23 receptor (IL-23R), facilitating Th17 differentiation. At high concentrations, TGF inhibits IL-23R, which is beneficial to Foxp3+ Tregs, thereby inhibiting the function of ROR γT cells. In contrast, IL-21 and IL-23 reduce Foxp3^+^-mediated inhibition of RORγT, thereby promoting Th17 differentiation.

TGFβ constitutes a key factor in the differentiation of regulatory T cells (Tregs) and Th17 cells. TGFβ is non-redundantly required for the generation of Tregs (28) but essential for the development of Th17 cells (29). IL-1β can take the place of TGFβ in IL-6-mediated generation of Th17 cells (30). In the absence of pro-inflammatory signals, such as IL-6 produced by microbial-activated dendritic cells (DCs) or IL-21 secreted by IL-6-stimulated T cells, priming of naïve CD4+ T cells in an environment rich in TGFβ promotes the development of iTregs (31) in response to antigens. In contrast, activation in an environment wherein both TGFβ and IL-6 are available promotes Th17 development, at least at mucosal sites (32). At low concentrations, TGFβ synergizes with IL-6 and IL-21 to increase IL-23 receptor (IL-23R) expression, favoring Th17 differentiation (33, 34), whereas at high concentrations, TGFβ represses IL-23R and favors Foxp3+ Tregs, which in turn restrains RORγt function (35). In contrast, the amelioration of IL-21 and IL-23 toward Foxp3-mediated inhibition of RORγt facilitates Th17 differentiation (Figure 2).

In a controlled clinical trial, altered methylation of interleukin (IL-6) and transforming growth factor β1 (TGF-β1) was detected. The logistic regression analysis showed that a combination of 14 CPGs in TGF-β1 and 4 CPGs in IL-6 provides a new way to identify children with CD and that CPGs, and the proximal fragment of the promoter of the TGF-β1 gene, discriminated quite accurately between children with UC and controls. The results of this controlled trial suggest that a combination of DNA methylation of TGF-β1 and IL-6 could provide very high accuracy in distinguishing between CD and control patients and that further expansion of the sample content could detect even more accurate variations. This provides a new way of diagnosing and classifying children with VEO-IBD (36).

### T and B Lymphocytes and Complex Function Defects

Loss of LRBA function mutations causes multiple defects in the immune cell population, which ultimately leads to the VEO-IBD phenotype (37). Arp2/3 and CARMIL2, also known as RLTPR, regulate the cytoskeleton, endocytosis, and cell migration by controlling actin polymerization (38). This study noted that CARMIL2 deficiency can produce an IBD-like phenotype, which may be related to the significant decrease in Foxp3+ Tregs in patients with CARMIL2 deficiency. Moreover, providing another important molecular example for PID to show the characteristics of VEO-IBD, this study placed great emphasis on the important role of CARMIL2-mediated immunity in regulation of the balance of the intestinal environment. The results of this study also suggested that the lack of a serious autoimmune phenotype in CARMIL2 deficiency may be caused by the deficiency of memory T cell differentiation and CD28-mediated dysfunction of effector T cell activation, but further research on CARMIL 2 is required to confirm this hypothesis.

Based on the relevant research results, we have summarized the existing and relatively comprehensive research conclusions, providing intuitive and useful conclusions (Table 1).

**Table 1:** List of gene mutations associated with monogenic VEO-IBD and IBD-like colitis.

**Genes**	**Clinical syndromes**	**Studies**
***Immune dysregulation***		
NOD2	Susceptibility gene	Travassos et al. (6)
ATG16L1	Susceptibility gene	Homer et al. (39)
TRIM22	NOD2 signaling defects	Li et al. (40)
IL-10	Neonatal or infantile VEO-IBD	Kotlarz et al. (24)
IL-10RA	Neonatal or infantile VEO-IBD	Glocker et al. (23)
IL-10RB	Neonatal or infantile VEO-IBD	Glocker et al. (23)
FOXP3	IPEX	Torgerson and Ochs (41)
***Hyperinflammatory and autoimmune disorders***		
XIAP	X-linked lymphoproliferative syndrome 2	Latour and Aguilar (42)
SLC11A1	Susceptibility gene	Sechi et al. (43)
CTLA4	Autoimmune lymphoproliferative syndrome	Kuehn et al. (44)
PLCG2	Autoinflammation and PLCγ2- associated antibody deficiency, and immune dysregulation (APLAID)	Zhou et al. (45)
STAT3	Multisystem autoimmune disease	Duerr et al. (46)
IL23R	Susceptibility gene	Duerr et al. (46)
CCR6	Susceptibility gene	Duerr et al. (46)
TNFSF15	Susceptibility gene	Duerr et al. (46)
Shp2	Susceptibility gene	Xiao et al. (47)
miR-320a	CD and UC	Fasseu et al. (11)
let-7c	CD and UC	Banerjee et al. (48)
***T cell, B cell, and complex function defects***		
LRBA	CVID 8	Alangari et al. (37)
ZAP70	ZAP70 deficiency	Chan et al. (49)
WAS	Wiscott-Aldrich syndrome	Catucci et al. (50)
CARMIL2	VEO-IBD, SCID	Magg et al. (38)
RAG2	Omenn syndrome	Kelsen et al. (51)
RAG1	Omenn syndrome	Villa et al. (52)
DCLRE1C/ARTEMIS95	Omenn syndrome	Villa et al. (52)
MALT1	SCID	Punwani et al. (53)
DOCK8	Hyper immunoglobulin E syndrome	Sanal et al. (54)
CD40LG	Hyper immunoglobulin M syndrome	Levy et al. (55)
AICDA	Hyper immunoglobulin M syndrome	Quartier et al. (56)
***Epithelial barrier function defects***		
COL7A1	Dystrophic epidermolysis bullosa	Zimmer et al. (57)
ADAM17	ADAM17 deficiency	Chalaris et al. (58)
IKBKG	X-linked ectodermal immunodeficiency (NEMO)	Karamchandani-Patel et al. (59)
FERMT1	Kindler syndrome	Sadler et al. (60)
TTC7A	TTC7A deficiency	Avitzur et al. (61)
GUCY2	Familial diarrhea	Uhlig (4)
CDKN2B-AS11	UC	Rankin et al. (21)
CircRNA_103765	IBD	Ye et al. (15)
Claudin2	IBD	Zeissig et al. (62)
HuR	Colitis	Pott et al. (18)
miR-126	IBD	Chen et al. (12)
***Others***		
FUT2	Susceptibility gene	McGovern et al. (63)

### Epithelial Barrier Function Defects

Villous blunting or atrophy barely occurs in adult IBD coteries but it occurs in as many as 20% of VEO-IBD coteries (51). Junctional adhesion molecule-A (JAM-A) constitutes a key structure of tight junctions and it is essential for the control of cell migration into the underlying tissues. Moreover, studies of CD tissue specimens revealed a loss of epithelial JAM-A expression (64). In line with these findings, decreased levels of other tight junction proteins, such as claudins, were observed in CD (62).

Defects in intestinal epithelial barrier function can be involved in VEO-IBD processes, including loss-of-function mutations in ADAM17 resulting in ADAM17 deficiency (65), IKBKG (encoding NEMO) mutation producing X-linked ectodermal dysplasia and immunodeficiency (59), and COL7A1 mutation causing dystrophic epidermolysis bullos (57). FERMT1 mutation results in Kindler syndrome (66), and TTC7A (61) or gain-of-function mutations in GUCY2 cause familial diarrhea (4, 67).

## Intestinal Environmental Factors

It has been proven that the interaction between the gut microbiota, metabolites, and the gut immune system is essential for maintaining a healthy gut (68). Specific alterations in the composition and function of the gut microbiota may be used as microbial biomarkers for the diagnosis of IBD, disease activity, response to therapy, and prediction of outcomes.

For newly diagnosed children with IBD, an increase in the number of *Proteobacteria* in the intestinal microbiota and a decrease in the number of *Faecalibacterium prausnitzii* appear to be associated with a complex disease phenotype and a subsequent need for biological therapy or surgery (69). The most common finding was an increase in adherent-aggressive *Escherichia coli* in the gut of IBD patients. This infectious agent can adhere to and cross the intestinal mucus barrier and invade the upper intestinal cortex. Second, compared with non-gastroenteritis patients, Salmonella fecal culture-positive gastroenteritis patients were significantly associated with an increased risk of new UC and CD. The most recent national case–control study from Sweden also demonstrated a positive association between a Salmonella diagnosis and the likelihood of IBD, and this study found that *Clostridium difficile* was associated with higher rates of UC and CD (70). Additionally, the latest and most comprehensive meta-analysis found a 57% lower risk of *Helicobacter pylori* exposure and inflammatory bowel disease, including CD and UC (71), and a meta-analysis that included only Asian studies reported consistent results (72). This protective association may be mediated, at least in part, by the specific components of *H. pylori* strains through immune regulation.

In addition to bacteria, some studies have found that VEO-IBD is also linked to enteroviruses. In a study of enterovirus, norovirus G-I, norovirus G-II, rotavirus, astrovirus, and sand wave virus RNA in fecal samples from 33 children with IBD and 17 children without IBD, viral RNA was detected only in children without IBD (3% vs. 0%) (73). Given changes in the intestinal microbiota in IBD patients, current clinical guidelines recommend testing for *C. difficile* in all IBD patients with exacerbating or newly emerging diarrhea and testing for cytomegalovirus in severely active IBD patients, especially when steroids and medications are used together to treat refractory disease.

Patients with CD exhibit significant differences in their gut metabolome, including lower concentrations of short-chain fatty acids (74, 75), higher concentrations of amino acids (75), and a dysregulated bile acid composition, including higher concentrations of conjugated bile acids and lower concentrations of secondary bile acids (76). In a cohort study, a strong association was found between an increase in the number of antibiotic prescriptions in the first year of life and the onset of IBD in childhood. In this study, the use of antibiotics, particularly in infancy, could lead to changes in the gut microbiota, suggesting they have a vital role in development of the immune system (77).

## Therapeutic Methods

### Medications

The standard therapeutic choices for VEO-IBD include 5-ASA, steroids, immunomodulators (6MP, azathioprine, methotrexate), and anti-TNF antibodies. At present, there are few studies that have been carried out in the pediatric patient population, and the relevant clinical data are insufficient, most of which are similar to polygenic IBD. It is more important to understand the data on drug-related dosage and adverse reactions (Table 2). Among the available medications, infliximab, vedolizumab, and ustekinumab are used in the treatment of VEO-IBD.

**Table 2:** Dose, interval, adverse reactions to medications for pediatric and VEO-IBD.

**Therapy**		**Therapeutic doses**	**The time interval**	**Adverse effects**
Exclusive enteral nutrition therapy		Starting from 10–20 ml/(kg*d), the speed of increasing 10–20 ml/(kg*d)	Total treatment: 6 weeks, then 2-week course of EEN tapering and gradual introduction of a habitual diet	Anatomical extension, perianal disease, stricturing behavior, diarrhea, vomiting, constipation, dehydration, feeding tube blockage, dyspnea and hypoxia, aspiration pneumonia.
Ustekinumab		90 mg	Every 8 weeks/every 4 weeks	Arthralgia, skin eruption, cough, spondylarthritis, cardiovascular disease, infection, malignant tumor, headache.
Vedolizumab		300 mg	0, 2 and 6 weeks, then every 8 weeks	Nausea, headache, malaise, spondylarthritis, bronchitis, liver function injury, fever, fatigue, back pain, limb pain, rash, pruritus, progressive multifocal leukoencephalopathy, infusion related reactions and hypersensitivity reactions.
Anti-TNF	Infliximab	5 mg/kg	0, 2 and 6 weeks, then every 8 weeks	(1) Acute infusion reactions: urticaria, fever, dyspnea, etc. (2) Delayed response: arthralgia, fever, rash, edema, and headache.
	Adalimumab	Induction period of 4 weeks, subcutaneous immunization Standard dose: 20 mg/kg or 40 mg/kg every week. Low dose: 10 mg/kg or 20 mg/kg every week.	<40 kg: 0 week, 80 mg. 2 week, 40 mg. >40 kg:0 week, 160 mg. 2 week, 80 mg.	(3) Infectious complications: urinary tract infection, pneumonia, cellulitis, mastitis, influenza and tuberculosis. (4) Cardiovascular system response. (5) Reactions: multiple sclerosis, paresthesia, and seizures. Malignant neoplasms and lupus-like syndrome.
	Golimumab	2 mg/kg	0, 4 weeks, then every 8 weeks	
CTLA4 agonists	Abatacept	<75 kg, 10 mg/kg ≥75 kg, 750 mg the maximum dose cannot exceed 1000 mg	0, 2, and 4 weeks, then every 4 weeks	(1) Discoid lupus, cutaneous vasculitis, erythema nodosum, skin infections, skin tumors. (2) Conditional infections: pathogenic pneumonia, sepsis. (3) Hematological toxicity. (4) Pulmonary sarcoidosis. (5) Tuberculous uveitis, eye pigment layer inflammation.
	Ipilimumab	3 mg/kg	Every 3 weeks	(1) Common adverse effects: insomnia, joint pain. (2) Serious adverse effects: oliguria, hematuresis, diarrhea, stomachache, cough, chest pain or wheezing, fatigue, memory problems, hallucinations, seizures, or neck stiffness, diureses weight loss, sweat, constipation, depression.

Monoclonal antibodies against tumor necrosis factor α(TNF-α), such as infliximab (IFX) or adalimumab (ADA), are safe and effective in inducing and maintaining remission in moderate-to-severe pediatric Crohn's disease (CD), and ulcerative colitis (UC) patients (78, 79). Based on the experience of a tertiary center in Japan, IFX treatment seems to be more effective for non-ulcerative colitis type (NUCT) and non-ulcerative colitis type without perianal disease (NUC-NPD) patients, and it seems that their height and weight are improved after treatment (80). However, the use of TNF inhibitors is limited, even among TNF responders, because of systemic side effects, including immunosuppression and cardiotoxicity. In addition, up to one-third of patients do not respond to TNF-α antagonist therapy, and ~20% of primary responders may experience response loss each year (81, 82). Therefore, in recent years, the FDA has approved some bio-similars ofTNF-α antagonists for the treatment ofVEO-IBD. The biosimilar agent infliximab is an immunoglobulin G(IgG) anti-TNF-α monoclonal antibody that binds to soluble and transmembrane forms of TNF-α, which can further impede its interaction with the TNF receptors TNFR1 (P55) and TNFR2 (P75) on the surface of target cells. Therefore, it can be used to treat pediatric IBD (83, 84). Nonetheless, the biosimilar adalimumab has not yet been approved for pediatric IBD (85).

Vedolizumab (VDZ) is a humanized monoclonal antibody that specifically identifies lymphocyte integrin 4β7 receptors and prevents them from migrating from the blood vessels into the intestinal mucosa, thereby reducing the flow of white blood cells into inflammatory tissues. As an intestinal selective anti-integrin drug, it has been reported to have a low risk of infection (86, 87). In the first study of VDZ in children with VEO-IBD, this anti-integrin agent was shown to be safe and effective in the study population. Similarly, in the Porto group study, 16 pediatric patients found VDZ was safe and well-tolerated−1 developed upper respiratory tract infection (6.3%), and two developed joint pain (12.5%) (88). Conrad et al. also evaluated VDZ for severe IBD in children with similar results (89). Another multicenter study published in 2016 demonstrated the efficacy and safety of VDZ in the pediatric population (90). VDZ is not approved for pediatric patients but has demonstrated clinical efficacy for pediatric IBD. Its remission rates of UC and CD are 76 and 42%, respectively (89, 90).

Ustekinumab, a therapeutic human IgG1 monoclonal antibody (mAb) targeting the interleukin (IL)-12/IL-23 shared p40 subunit, is approved in adolescents (12 years of age and older) for the treatment of moderate and severe psoriasis, as well as for the treatment of adult celiac disease and UC (91)while celiac disease currently has gluten-free diet as the only therapy. In a multicenter prospective cohort of children, the effectiveness of ustekinumab in treating refractory UC was demonstrated (92), which is similar to the results of a retrospective study of pediatric IBD, suggesting that ustekinumab is effective and safe in children with IBD (93). Some case reports suggest that 50% of children with IBD have a clinical response to ustekinumab (94, 95). In a cohort of pediatric patients with CD, patients using ustekinumab had significant improvements in their abbreviated pediatric CD activity index (aPCDAI) scores, clinical remission rates, albumin, and hematocrit, and 89.5% of patients had no significant adverse events (96). The use of off-label drugs is increasing in children with IBD and generally they are being reported as safe and effective (93).

From limited evidence, dual biotherapy may be a safe option for patients with refractory IBD who have failed multiple biotherapies and for managing the extra-intestinal presentation of IBD (97). A cohort of refractory pediatric IBD reported the effectiveness and safety of dual biologics or a combination of biologics and JAK inhibitors (98). In a case series and review of the literature, eight children received a combination of infliximab and vedolizumab, and five children received a combination of infliximab and ustekinumab, which shows combining biological agents to be safe and beneficial in selected patients (99). However, larger studies are required to confirm the preliminary safety data that were observed.

Ruxolitinib, a selective JAK1/2 inhibitor, was found in a single-center retrospective study of patients with refractory VEO-IBD with AIP to be primarily used for dual therapy when complete remission was not achieved with primary therapy. All patients in this study showed clinical improvement and did not require complete parenteral nutrition or steroids. Other potential benefits of ruxolitinib included a lack of immunogenicity, a rapid onset of action, and a short half-life. In addition, ruxolitinib can be administered entirely with sufficient enteral absorption to achieve a clinical response in a cohort with severe intestinal disease. However, this study still has limitations and could not determine whether ruxolitinib will be effective or safe in general use (100).

### Exclusive Enteral Nutrition Therapy

Exclusive enteral nutrition therapy (EEN) is the preferred treatment for European VEO-IBD patients. In a propensity score matching cohort analysis of children with Crohn's disease induced by total enteral nutritionor glucocorticoids (CSs) (101), EEN, and CSs were found to be equally effective in inducing remission. Through a central retrospective analysis, EEN was found to be more effective than CSs in improving nutritional status and growth recovery, with relatively few side effects. More importantly, EEN can achieve mucosal healing (MH), which is the target of CD treatment. When applied in an early stage, MH reduces the incidence of hospitalization, surgical resection, and fistula formation, providing a new pattern for the treatment of very early inflammatory bowel disease. Some data show that the intestinal flora, amino acids, and fecal metabolites of CD patients have significant changes before and after EEN treatment (102), providing a biochemical detection method for judging the efficacy of EEN.

### Hematopoietic Stem Cell Transplantation

Due to events such as severe or opportunistic infections and malignancies associated with biologic methods, stem cell transplantation has entered clinical trials as a more permanent treatment for IBD. Allogeneic hematopoietic stem cell transplantation (allo-HSCT) is an established therapeutic option for VEO-IBD. In a retrospective investigation of autologous hematopoietic stem cell transplantation for CD, MSC therapy may be an alternative to endovenous and fistula treatment (103). In terms of IPEX and IL-10 signaling deficits, HSCT has been shown to improve colitis and gastrointestinal fistula (104, 105). VEO-IBD patients with IL-10R deficiency can also be cured by allo-HSCT (23, 106). However, 11 patients with transplanted IL-10 and IL-10R deficiency showed a very high frequency of primary graft rejection (3/11), and these data suggest that patients with either IL-10 or IL-10R deficiency need to have their transplant regimen adjusted to reduce the risk of rejection (105, 106). Therefore, the use of post-transplant cyclophosphamide and bone marrow transplantation with T cell-identical cells have been considered potential therapies for patients with IL-10R deficiency (107). Similarly, studies have shown that allogeneic hematopoietic stem cell transplantation can successfully treat XIAP-deficient idiopathic colitis with specific conditioning regimens (108). For patients with a refractory IBD phenotype and an increased risk of mortality due to XIAP deficiency, HSCT should be considered as early as possible, as it can address their risk of intestinal inflammation and the development of life-threatening hemophilic lymphocytosis (109). However, HSCT is not effective for all cases of VEO-IBD. IBD lacking NEMO or TTC7A cannot be improved after HSCT and it may even worsen (110, 111). Therefore, we believe that the application of therapeutic HSCT in certain conditions is promising, but it should always be personalized.

Bone marrow MSCs can promote wound healing and tissue regeneration by secreting TGF-β and fibroblast growth factor. This property offers a new approach to the treatment of CD with fistulae.

### Surgery

Despite recent advances in treatments, surgery still plays an important role in the management of VEO-IBD. Although surgery cannot cure VEO-IBD, in some cases, it can help resolve acute complications and maintain remission, allowing disease-free intervals, and nutritional recovery (112), and has a huge impact on physical and mental development (113). The main indications for surgery in CD are unresponsive and refractory to maximum medical treatment, fistula, perforation, stricturing disease, and severe perianal disease. Meanwhile, acute indications for UC surgery also include toxic megacolon, which is rare in children. A systematic review noted that surgical rates for CD ranged from 10 to 72%, while colectomy rates for UC ranged from 0 to 50% (113). Minimally invasive surgery has also been used for the radical treatment of CD and UC since 2002. In recent years, robotic surgery, a single-hole approach and minimally invasive treatment of perianal fistula CD have been adopted (114).

### Other Treatments

Two recent randomized controlled trials (RCTs) provided additional insight by suggesting that rebuilding the intestinal microbiota composition through fecal microflora transplantation (FMT) can improve UC activity in this patient subgroup (115, 116). Although a few nonrandomized control studies have been performed in older children (youngest child 7 years old) (117), the efficacy of FMT for VEO-IBD is unclear.

The use of immunosuppressive drugs in the treatment regimen increases the risk of infectious diseases and infection-related complications in children with IBD. Therefore, vaccination to prevent related infections is an important aspect of long-term care of this disease.

## Conclusion

Current studies show that the pathogenesis of VEO-IBD includes genetic factors, immune molecular factors, and changes in the intestinal environment. With research progress on the susceptibility genes of IBD, the localization of the susceptibility genes of IBD helps identify and distinguish the disease phenotype, track the clinical progress, and ultimately develop new targeted therapies. However, due to the lack of clinical follow-up data in VEO-IBD children, there are still great challenges in terms of the drug efficacy and the research and development (R&D) of new drugs. It is not enough to draw lessons from the adult treatment experience alone. In the future, rapid diagnosis and management of children should be carried out, diagnosis, and treatment criteria based on genetic abnormalities should be established, and a clinical database should be expanded. We need to establish control groups and explore the influence of environmental factors on the incidence, treatment and prognosis of VEO-IBD. Finally, a precision medicine model needs to be achieved, namely, individualized treatment for VEO-IBD children.

## Author Contributions

Q-QL and H-HZ drafted the article and approved the final manuscript as submitted.

## Funding

This study was supported by the High-Level Personnel Program of Guangdong Provincial People's Hospital (2021DFJH0008/KY012021458), Starting Program for National Natural Science Foundation of China at Guangdong Provincial People's Hospital (8207034250), National Natural Science Foundation of China (NSFC, No. 81300370), China Postdoctoral Science Foundation (CPSF, No. 2018T110855 of Special Support Program and No. 2017M622650 of General Support Program), Natural Science Foundation of Guangdong (NSFG, No. 2018A030313161), Southern Medical University (12440000771868596D), S-XD is a supervisor at the Second Clinical School of Southern Medical University and an adjunct associate professor at Southern Medical University, and supervised Q-QL and H-HZ in New Insights and Advances in Pathogenesis and Treatment of Very Early Onset Inflammatory Bowel Disease.

## Conflict of Interest

The authors declare that the research was conducted in the absence of any commercial or financial relationships that could be construed as a potential conflict of interest.

## Publisher's Note

All claims expressed in this article are solely those of the authors and do not necessarily represent those of their affiliated organizations, or those of the publisher, the editors and the reviewers. Any product that may be evaluated in this article, or claim that may be made by its manufacturer, is not guaranteed or endorsed by the publisher.

## References

[1] Miller TL Lee D Giefer M Wahbeh G Suskind DL. Nutritional therapy in very early-onset inflammatory bowel disease: a case report. Digest Dis Sci. (2017) 62:2196–200. 10.1007/s10620-017-4616-9 28551707

[2] Kelsen JR Sullivan KE Rabizadeh S Singh N Snapper S Elkadri A. North american society for pediatric gastroenterology, hepatology, and nutrition position paper on the evaluation and management for patients with very early-onset inflammatory bowel disease. J Pediatr Gastroenterol Nutr. (2020) 70:389–403. 10.1097/MPG.0000000000002567 32079889 PMC12024488

[3] Zhang YZ Li YY. Inflammatory bowel disease: pathogenesis. World J Gastroenterol. (2014) 20:91–9. 10.3748/wjg.v20.i1.91 24415861 PMC3886036

[4] Uhlig HH. Monogenic diseases associated with intestinal inflammation: implications for the understanding of inflammatory bowel disease. Gut. (2013) 62:1795–805. 10.1136/gutjnl-2012-303956 24203055

[5] Parkhouse R Monie TP. Dysfunctional Crohn's disease-associated NOD2 polymorphisms cannot be reliably predicted on the basis of RIPK2 binding or membrane association. Front Immunol. (2015) 6:521. 10.3389/fimmu.2015.00521 26500656 PMC4597273

[6] Travassos LH Carneiro LA Ramjeet M Hussey S Kim YG Magalhaes JG. Nod1 and Nod2 direct autophagy by recruiting ATG16L1 to the plasma membrane at the site of bacterial entry. Nat Immunol. (2010) 11:55–62. 10.1038/ni.1823 19898471

[7] Cooney R Baker J Brain O Danis B Pichulik T Allan P. NOD2 stimulation induces autophagy in dendritic cells influencing bacterial handling and antigen presentation. Nat Med. (2010) 16:90–7. 10.1038/nm.2069 19966812

[8] Jensen MD Andersen RF Christensen H Nathan T Kjeldsen J Madsen JS. Circulating microRNAs as biomarkers of adult Crohn's disease. Eur J Gastroenterol Hepatol. (2015) 27:1038–44. 10.1097/MEG.0000000000000430 26230660

[9] Batra SK Heier CR Diaz-Calderon L Tully CB Fiorillo AA van den Anker J. Serum miRNAs are pharmacodynamic biomarkers associated with therapeutic response in pediatric inflammatory bowel disease. Inflamm Bowel Dis. (2020) 26:1597–606. 10.1093/ibd/izaa209 32793975 PMC7500519

[10] Paterson MR Kriegel AJ. MiR-146a/b: a family with shared seeds and different roots. Physiol Genomics. (2017) 49:243–52. 10.1152/physiolgenomics.00133.2016 28213571 PMC5407182

[11] Fasseu M Treton X Guichard C Pedruzzi E Cazals-Hatem D Richard C. Identification of restricted subsets of mature microRNA abnormally expressed in inactive colonic mucosa of patients with inflammatory bowel disease. PLoS ONE. (2010) 5: e13160. 10.1371/journal.pone.0013160 20957151 PMC2950152

[12] Chen T Xue H Lin R Huang Z. MiR-126 impairs the intestinal barrier function via inhibiting S1PR2 mediated activation of PI3K/AKT signaling pathway. Biochem Biophys Res Commun. (2017) 494:427–32. 10.1016/j.bbrc.2017.03.043 28302479

[13] Li X Yang L Chen LL. The biogenesis, functions, and challenges of circular RNAs. Mol Cell. (2018) 71:428–42. 10.1016/j.molcel.2018.06.034 30057200

[14] Yin J Hu T Xu L Li P Li M Ye Y. Circular RNA expression profile in peripheral blood mono-nuclear cells from Crohn disease patients. Medicine (Baltimore). (2019) 98:e16072. 10.1097/MD.0000000000016072 31261517 PMC6617429

[15] Ye Y Zhang L Hu T Yin J Xu L Pang Z. CircRNA_103765 acts as a pro-inflammatory factor via sponging miR-30 family in Crohn's disease. Sci Rep. (2021) 11:565. 10.1038/s41598-020-80663-w 33436852 PMC7804428

[16] Qiao YQ Cai CW Shen J Zheng Q Ran ZH. Circular RNA expression alterations in colon tissues of Crohn's disease patients. Mol Med Rep. (2019) 19:4500–6. 10.3892/mmr.2019.10070 30896837

[17] Giammanco A Blanc V Montenegro G Klos C Xie Y Kennedy S. Intestinal epithelial HuR modulates distinct pathways of proliferation and apoptosis and attenuates small intestinal and colonic tumor development. Cancer Res. (2014) 74:5322–35. 10.1158/0008-5472.CAN-14-0726 25085247 PMC4167566

[18] Pott J Kabat AM Maloy KJ. Intestinal epithelial cell autophagy is required to protect against TNF-induced apoptosis during chronic colitis in mice. Cell Host Microbe. (2018) 23:191–202. 10.1016/j.chom.2017.12.017 29358084

[19] Holdt LM Stahringer A Sass K Pichler G Kulak NA Wilfert W. Circular non-coding RNA ANRIL modulates ribosomal RNA maturation and atherosclerosis in humans. Nat Commun. (2016) 7:12429. 10.1038/ncomms12429 27539542 PMC4992165

[20] Holdt LM Hoffmann S Sass K Langenberger D Scholz M Krohn K. Alu elements in ANRIL non-coding RNA at chromosome 9p21 modulate atherogenic cell functions through trans-regulation of gene networks. PLoS Genet. (2013) 9:e1003588. 10.1371/journal.pgen.1003588 23861667 PMC3701717

[21] Rankin CR Lokhandwala ZA Huang R Pekow J Pothoulakis C Padua D. Linear and circular CDKN2B-AS1 expression is associated with inflammatory bowel disease and participates in intestinal barrier formation. Life Sci. (2019) 231:116571. 10.1016/j.lfs.2019.116571 31207308 PMC6897550

[22] Fiorentino DF Bond MW Mosmann TR. Two types of mouse T helper cell. IV. Th2 clones secrete a factor that inhibits cytokine production by Th1 clones. J Exp Med. (1989) 170: 2081–95. 10.1084/jem.170.6.2081 2531194 PMC2189521

[23] Glocker EO Kotlarz D Boztug K Gertz EM Schaffer AA Noyan F. Inflammatory bowel disease and mutations affecting the interleukin-10 receptor. N Engl J Med. (2009) 361:2033–45. 10.1056/NEJMoa0907206 19890111 PMC2787406

[24] Glocker EO Frede N Perro M Sebire N Elawad M Shah N. Infant colitis–it's in the genes. Lancet. (2010) 376:1272. 10.1016/S0140-6736(10)61008-2 20934598

[25] Shouval DS Ouahed J Biswas A Goettel JA Horwitz BH Klein C. Interleukin 10 receptor signaling: master regulator of intestinal mucosal homeostasis in mice and humans. Adv Immunol. (2014) 122:177–210. 10.1016/B978-0-12-800267-4.00005-5 24507158 PMC4741283

[26] Wolk K Sabat R. Interleukin-22: A novel T- and NK-cell derived cytokine that regulates the biology of tissue cells. Cytokine Growth Factor Rev. (2006) 17:367–80. 10.1016/j.cytogfr.2006.09.001 17030002

[27] Franke A Balschun T Karlsen TH Sventoraityte J Nikolaus S Mayr G. Sequence variants in IL10, ARPC2 and multiple other loci contribute to ulcerative colitis susceptibility. Nat Genet. (2008) 40:1319–23. 10.1038/ng.221 18836448

[28] Nakamura K Kitani A Strober W. Cell contact-dependent immunosuppression by CD4(+)CD25(+) regulatory T cells is mediated by cell surface-bound transforming growth factor beta. J Exp Med. (2001) 194:629–44. 10.1084/jem.194.5.629 11535631 PMC2195935

[29] Das J Ren G Zhang L Roberts AI Zhao X Bothwell AL. Transforming growth factor beta is dispensable for the molecular orchestration of Th17 cell differentiation. J Exp Med. (2009) 206:2407–16. 10.1084/jem.20082286 19808254 PMC2768861

[30] Ghoreschi K Laurence A Yang XP Tato CM McGeachy MJ Konkel JE. Generation of pathogenic T(H)17 cells in the absence of TGF-beta signalling. Nature. (2010) 467:967–71. 10.1038/nature09447 20962846 PMC3108066

[31] Bettelli E Carrier Y Gao W Korn T Strom TB Oukka M. Reciprocal developmental pathways for the generation of pathogenic effector TH17 and regulatory T cells. Nature. (2006) 441:235–8. 10.1038/nature04753 16648838

[32] Hu W Troutman TD Edukulla R Pasare C. Priming microenvironments dictate cytokine requirements for T helper 17 cell lineage commitment. Immunity. (2011) 35:1010–22. 10.1016/j.immuni.2011.10.013 22137454 PMC3246047

[33] Korn T Bettelli E Gao W Awasthi A Jager A Strom TB. IL-21 initiates an alternative pathway to induce proinflammatory T(H)17 cells. Nature. (2007) 448:484–7. 10.1038/nature05970 17581588 PMC3805028

[34] Nurieva R Yang XO Martinez G Zhang Y Panopoulos AD Ma L. Essential autocrine regulation by IL-21 in the generation of inflammatory T cells. Nature. (2007) 448:480–3. 10.1038/nature05969 17581589

[35] Zhou L Lopes JE Chong MM Ivanov II Min R Victora GD. TGF-beta-induced Foxp3 inhibits T(H)17 cell differentiation by antagonizing RORgammat function. Nature. (2008) 453:236–40. 10.1038/nature06878 18368049 PMC2597437

[36] Mitsuyama K Sata M Rose-John S. Interleukin-6 trans-signaling in inflammatory bowel disease. Cytokine Growth Factor Rev. (2006) 17:451–61. 10.1016/j.cytogfr.2006.09.003 17045835

[37] Alangari A Alsultan A Adly N Massaad MJ Kiani IS Aljebreen A. LPS-responsive beige-like anchor (LRBA) gene mutation in a family with inflammatory bowel disease and combined immunodeficiency. J Allergy Clin Immunol. (2012) 130:481–8. 10.1016/j.jaci.2012.05.043 22721650 PMC3582381

[38] Magg T Shcherbina A Arslan D Desai MM Wall S Mitsialis V. CARMIL2 deficiency presenting as very early onset inflammatory bowel disease. Inflamm Bowel Dis. (2019) 25:1788–95. 10.1093/ibd/izz103 31115454 PMC6799948

[39] Homer CR Richmond AL Rebert NA Achkar JP McDonald C. (2010). ATG16L1 and NOD2 interact in an autophagy-dependent antibacterial pathway implicated in Crohn's disease pathogenesis. Gastroenterol. (1641) 139:1630–41. 10.1053/j.gastro.2010.07.006 20637199 PMC2967588

[40] Li Q Lee CH Peters LA Mastropaolo LA Thoeni C Elkadri A. Variants in TRIM22 that affect NOD2 signaling are associated with very-early-onset inflammatory bowel disease. Gastroenterol. (2016) 150:1196–207. 10.1053/j.gastro.2016.01.031 26836588 PMC4842103

[41] Torgerson TR Ochs HD. Immune dysregulation, polyendocrinopathy, enteropathy, X-linked: Forkhead box protein three mutations and lack of regulatory T cells. J Allergy Clin Immunol. (2007) 120: 744–52. 10.1016/j.jaci.2007.08.044 17931557

[42] Latour S Aguilar C. XIAP deficiency syndrome in humans. Semin Cell Dev Biol. (2015) 39:115–23. 10.1016/j.semcdb.2015.01.015 25666262

[43] Sechi LA Gazouli M Sieswerda LE Molicotti P Ahmed N Ikonomopoulos J. Relationship between Crohn's disease, infection with Mycobacterium avium subspecies paratuberculosis, and SLC11A1 gene polymorphisms in Sardinian patients. World J Gastroenterol. (2006) 12:7161–4. 10.3748/wjg.v12.i44.7161 17131479 PMC4087778

[44] Kuehn HS Ouyang W Lo B Deenick EK Niemela JE Avery DT. Immune dysregulation in human subjects with heterozygous germline mutations in CTLA4. Science. (2014) 345:1623–7. 10.1126/science.1255904 25213377 PMC4371526

[45] Zhou Q Lee GS Brady J Datta S Katan M Sheikh A. A hypermorphic missense mutation in PLCG2, encoding phospholipase Cgamma2, causes a dominantly inherited auto-inflammatory disease with immunodeficiency. Am J Hum Genet. (2012) 91:713–20. 10.1016/j.ajhg.2012.08.006 23000145 PMC3484656

[46] Duerr RH Taylor KD Brant SR Rioux JD Silverberg MS Daly MJ. A genome-wide association study identifies IL23R as an inflammatory bowel disease gene. Science. (2006) 314:1461–3. 10.1126/science.1135245 17068223 PMC4410764

[47] Xiao P Zhang H Zhang Y Zheng M Liu R Zhao Y. Phosphatase Shp2 exacerbates intestinal inflammation by disrupting macrophage responsiveness to interleukin-10. J Exp Med. (2019) 216:337–49. 10.1084/jem.20181198 30610104 PMC6363431

[48] Banerjee S Xie N Cui H Tan Z Yang S Icyuz M. MicroRNA let-7c regulates macrophage polarization. J Immunol. (2013) 190:6542–9. 10.4049/jimmunol.1202496 23667114 PMC3679284

[49] Chan AC Kadlecek TA Elder ME Filipovich AH Kuo WL Iwashima M. ZAP-70 deficiency in an autosomal recessive form of severe combined immunodeficiency. Science. (1994) 264:1599–601. 10.1126/science.8202713 8202713

[50] Catucci M Castiello MC Pala F Bosticardo M Villa A. Autoimmunity in wiskott-aldrich syndrome: an unsolved enigma. Front Immunol. (2012) 3:209. 10.3389/fimmu.2012.00209 22826711 PMC3399097

[51] Kelsen JR Russo P Sullivan KE. Early-onset inflammatory bowel disease. Immunol Allergy Clin North Am. (2019) 39:63–79. 10.1016/j.iac.2018.08.008 30466773 PMC6954002

[52] Villa A Notarangelo LD Roifman CM. Omenn syndrome: inflammation in leaky severe combined immunodeficiency. J Allergy Clin Immunol. (2008) 122:1082–6. 10.1016/j.jaci.2008.09.037 18992930

[53] Punwani D Wang H Chan AY Cowan MJ Mallott J Sunderam U. Combined immunodeficiency due to MALT1 mutations, treated by hematopoietic cell transplantation. J Clin Immunol. (2015) 35:135–46. 10.1007/s10875-014-0125-1 25627829 PMC4352191

[54] Sanal O Jing H Ozgur T Ayvaz D Strauss-Albee DM Ersoy-Evans S. Additional diverse findings expand the clinical presentation of DOCK8 deficiency. J Clin Immunol. (2012) 32:698–708. 10.1007/s10875-012-9664-5 22476911 PMC3732775

[55] Levy J Espanol-Boren T Thomas C Fischer A Tovo P Bordigoni P. Clinical spectrum of X-linked hyper-IgM syndrome. J Pediatr. (1997) 131:47–54. 10.1016/s0022-3476(97)70123-9 9255191

[56] Quartier P Bustamante J Sanal O Plebani A Debre M Deville A. Clinical, immunologic and genetic analysis of 29 patients with autosomal recessive hyper-IgM syndrome due to Activation-Induced Cytidine Deaminase deficiency. Clin Immunol. (2004) 110:22–9. 10.1016/j.clim.2003.10.007 14962793

[57] Zimmer KP Schumann H Mecklenbeck S Bruckner-Tuderman L. Esophageal stenosis in childhood: dystrophic epidermolysis bullosa without skin blistering due to collagen VII mutations. Gastroenterol. (2002) 122:220erol 10.1053/gast.2002.30428 11781296

[58] Chalaris A Gewiese J Paliga K Fleig L Schneede A Krieger K. ADAM17-mediated shedding of the IL6R induces cleavage of the membrane stub by gamma-secretase. Biochim Biophys Acta. (2010) 1803:234–45. 10.1016/j.bbamcr.2009.12.001 20026129

[59] Karamchandani-Patel G Hanson EP Saltzman R Kimball CE Sorensen RU Orange JS. Congenital alterations of NEMO glutamic acid 223 result in hypohidrotic ectodermal dysplasia and immunodeficiency with normal serum IgG levels. Ann Allergy Asthma Immunol. (2011) 107:50–6. 10.1016/j.anai.2011.03.009 21704885 PMC3177139

[60] Sadler E Klausegger A Muss W Deinsberger U Pohla-Gubo G Laimer M. Novel KIND1 gene mutation in Kindler syndrome with severe gastrointestinal tract involvement. Arch Dermatol. (2006) 142:1619–24. 10.1001/archderm.142.12.1619 17178989

[61] Avitzur Y Guo C Mastropaolo LA Bahrami E Chen H Zhao Z. Mutations in tetra-tricopeptide repeat domain 7A result in a severe form of very early onset inflammatory bowel disease. Gastroenterol. (2014) 146:1028–39. 10.1053/j.gastro.2014.01.015 24417819 PMC4002656

[62] Zeissig S Burgel N Gunzel D Richter J Mankertz J Wahnschaffe U. Changes in expression and distribution of claudin 2, 5, and 8 lead to discontinuous tight junctions and barrier dysfunction in active Crohn's disease. Gut. (2007) 56:61–72. 10.1136/gut.2006.094375 16822808 PMC1856677

[63] McGovern DP Jones MR Taylor KD Marciante K Yan X Dubinsky M. Fucosyltransferase 2 (FUT2) non-secretor status is associated with Crohn's disease. Hum Mol Genet. (2010) 19:3468–76. 10.1093/hmg/ddq248 20570966 PMC2916706

[64] Vetrano S Rescigno M Cera MR Correale C Rumio C Doni A. Unique role of junctional adhesion molecule-a in maintaining mucosal homeostasis in inflammatory bowel disease. Gastroenterology. (2008) 135:173–84. 10.1053/j.gastro.2008.04.002 18514073

[65] Blaydon DC Biancheri P Di WL Plagnol V Cabral RM Brooke MA. Inflammatory skin and bowel disease linked to ADAM17 deletion. N Engl J Med. (2011) 365:1502–8. 10.1056/NEJMoa1100721 22010916

[66] Ussar S Moser M Widmaier M Rognoni E Harrer C Genzel-Boroviczeny O. Loss of Kindlin-1 causes skin atrophy and lethal neonatal intestinal epithelial dysfunction. PLoS Genet. (2008) 4:e1000289. 10.1371/journal.pgen.1000289 19057668 PMC2585060

[67] Fiskerstrand T Arshad N Haukanes BI Tronstad RR Pham KD Johansson S. Familial diarrhea syndrome caused by an activating GUCY2C mutation. N Engl J Med. (2012) 366:1586–95. 10.1056/NEJMoa1110132 22436048

[68] Haberman Y Tickle TL Dexheimer PJ Kim MO Tang D Karns R. Pediatric Crohn disease patients exhibit specific ileal transcriptome and microbiome signature. J Clin Invest. (2014) 124:3617–33. 10.1172/JCI75436 25003194 PMC4109533

[69] Olbjorn C Cvancarova SM Thiis-Evensen E Nakstad B Vatn MH Jahnsen J. Fecal microbiota profiles in treatment-naive pediatric inflammatory bowel disease - associations with disease phenotype, treatment, and outcome. Clin Exp Gastroenterol. (2019) 12:37–49. 10.2147/CEG.S186235 30774408 PMC6362922

[70] Axelrad JE Olen O Askling J Lebwohl B Khalili H Sachs MC. Gastrointestinal infection increases odds of inflammatory bowel disease in a nationwide case-control study. Clin Gastroenterol Hepatol. (2019) 17:1311–22. 10.1016/j.cgh.2018.09.034 30389589

[71] Castano-Rodriguez N Kaakoush NO Lee WS Mitchell HM. Dual role of Helicobacter and Campylobacter species in IBD: a systematic review and meta-analysis. Gut. (2017) 66:235–49. 10.1136/gutjnl-2015-310545 26508508

[72] Wu XW Ji HZ Yang MF Wu L Wang FY. Helicobacter pylori infection and inflammatory bowel disease in Asians: a meta-analysis. World J Gastroenterol. (2015) 21:4750–6. 10.3748/wjg.v21.i15.4750 25914487 PMC4402325

[73] Kolho KL Klemola P Simonen-Tikka ML Ollonen ML Roivainen M. Enteric viral pathogens in children with inflammatory bowel disease. J Med Virol. (2012) 84:345–7. 10.1002/jmv.23193 22170557

[74] Treem WR Ahsan N Shoup M Hyams JS. Fecal short-chain fatty acids in children with inflammatory bowel disease. J Pediatr Gastroenterol Nutr. (1994) 18:159–64. 10.1097/00005176-199402000-00007 8014762

[75] Bjerrum JT Wang Y Hao F Coskun M Ludwig C Gunther U. Metabonomics of human fecal extracts characterize ulcerative colitis, Crohn's disease and healthy individuals. Metabolomics. (2015) 11:122–33. 10.1007/s11306-014-0677-3 25598765 PMC4289537

[76] Duboc H Rajca S Rainteau D Benarous D Maubert MA Quervain E. Connecting dysbiosis, bile-acid dysmetabolism, and gut inflammation in inflammatory bowel diseases. Gut. (2013) 62:531–9. 10.1136/gutjnl-2012-302578 22993202

[77] Canova C Ludvigsson JF Di Domenicantonio R Zanier L Barbiellini AC Zingone F. Perinatal and antibiotic exposures and the risk of developing childhood-onset inflammatory bowel disease: a nested case-control study based on a population-based birth cohort. Int J Environ Res Public Health. (2020) 17:2409. 10.3390/ijerph17072409 32252276 PMC7177699

[78] Hyams J Crandall W Kugathasan S Griffiths A Olson A Johanns J. Induction and maintenance infliximab therapy for the treatment of moderate-to-severe Crohn's disease in children. Gastroenterol. (2007) 132: 863–73. 10.1053/j.gastro.2006.12.003 17324398

[79] Hyams JS Lerer T Griffiths A Pfefferkorn M Stephens M Evans J. Outcome following infliximab therapy in children with ulcerative colitis. Am J Gastroenterol. (2010) 105:1430–430 10.1038/ajg.2009.759 20104217

[80] Takeuchi I Kaburaki Y Arai K Shimizu H Hirano Y Nagata S. Infliximab for very early-onset inflammatory bowel disease: a tertiary center experience in Japan. J Gastroenterol Hepatol. (2020) 35:593terol 10.1111/jgh.14836 31425641

[81] Gisbert JP Marin AC McNicholl AG Chaparro M. Systematic review with meta-analysis: the efficacy of a second anti-TNF in patients with inflammatory bowel disease whose previous anti-TNF treatment has failed. Aliment Pharmacol Ther. (2015) 41:613l The 10.1111/apt.13083 25652884

[82] Gisbert JP Panes J. Loss of response and requirement of infliximab dose intensification in Crohn's disease: a review. Am J Gastroenterol. (2009) 104:760erol 10.1038/ajg.2008.88 19174781

[83] Aboobacker S Al Aboud AM. Infliximab-abda. In: StatPearls. Treasure island, FL: StatPearls Publishing (2021). 30725744

[84] Fatima R Bittar K Aziz M. Infliximab. In: StatPearls. Treasure island, FL: StatPearls Publishing (2021). 29763197

[85] Ellis CR Azmat CE. Adalimumab. In: StatPearls. Treasure island, FL: StatPearls Publishing (2021).

[86] Ng SC Hilmi IN Blake A Bhayat F Adsul S Khan QR. Low frequency of opportunistic infections in patients receiving vedolizumab in clinical trials and post-marketing setting. Inflamm Bowel Dis. (2018) 24:2431–431:10.1093/ibd/izy153 30312414 PMC6185254

[87] Meserve J Aniwan S Koliani-Pace JL Shashi P Weiss A Faleck D. Retrospective analysis of safety of vedolizumab in patients with inflammatory bowel diseases. Clin Gastroenterol Hepatol. (2019) 17:1533–533:10.1016/j.cgh.2018.09.035 30268561 PMC6594363

[88] Fabiszewska S Derda E Szymanska E Osiecki M Kierkus J. Safety and effectiveness of vedolizumab for the treatment of pediatric patients with very early onset inflammatory bowel diseases. J Clin Med. (2021). 10:2997. 10.3390/jcm10132997 34279480 PMC8268556

[89] Conrad MA Stein RE Maxwell EC Albenberg L Baldassano RN Dawany N. Vedolizumab therapy in severe pediatric inflammatory bowel disease. Inflamm Bowel Dis. (2016) 22:2425–425:10.1097/MIB.0000000000000918 27598742

[90] Singh N Rabizadeh S Jossen J Pittman N Check M Hashemi G. Multi-Center experience of vedolizumab effectiveness in pediatric inflammatory bowel disease. Inflamm Bowel Dis. (2016) 22:2121–121 10.1097/MIB.0000000000000865 27542130

[91] Chavannes M Martinez-Vinson C Hart L Kaniki N Chao CY Lawrence S. Management of paediatric patients with medically refractory crohn's disease using ustekinumab: a multi-centred cohort study. J Crohns Colitis. (2019) 13:578oliti 10.1093/ecco-jcc/jjy206 30541021

[92] Dhaliwal J McKay HE Deslandres C Debruyn J Wine E Wu A. 1-year outcomes with ustekinumab therapy in infliximab-refractory paediatric ulcerative colitis: a multicentre prospective study. Aliment Pharmacol Ther. (2021) 53:1300–300 10.1111/apt.16388 33909911

[93] Dayan JR Dolinger M Benkov K Dunkin D Jossen J Lai J. Real world experience with ustekinumab in children and young adults at a tertiary care pediatric inflammatory bowel disease center. J Pediatr Gastroenterol Nutr. (2019) 69:61–7. 10.1097/MPG.0000000000002362 31058718 PMC7408448

[94] Bishop C Simon H Suskind D Lee D Wahbeh G. Ustekinumab in pediatric crohn disease patients. J Pediatr Gastroenterol Nutr. (2016) 63:348–51. 10.1097/MPG.0000000000001146 26854655

[95] Cameron FL Garrick V Russell RK. Ustekinumab in treatment of refractory paediatric crohn disease. J Pediatr Gastroenterol Nutr. (2016) 62:e30. 10.1097/MPG.0000000000000608 26910458

[96] Kim FS Patel PV Stekol E Ali S Hamandi H Heyman MB. Experience using ustekinumab in pediatric patients with medically refractory crohn disease. J Pediatr Gastroenterol Nutr. (2021) 73:610–4. 10.1097/MPG.0000000000003230 34415711 PMC8542638

[97] Haider M Lashner B. Dual targeted therapy for the management of inflammatory bowel disease. J Clin Gastroenterol. (2021) 55:661–6. 10.1097/MCG.0000000000001583 34238847

[98] Dolinger MT Spencer EA Lai J Dunkin D Dubinsky MC. Dual biologic and small molecule therapy for the treatment of refractory pediatric inflammatory bowel disease. Inflamm Bowel Dis. (2021) 27:1210–4. 10.1093/ibd/izaa277 33125058

[99] Olbjorn C Rove JB Jahnsen J. Combination of biological agents in moderate to severe pediatric inflammatory bowel disease: a case series and review of the literature. Paediatr Drugs. (2020) 22:409–16. 10.1007/s40272-020-00396-1 32378002 PMC7383034

[100] Rudra S Shaul E Conrad MA Patel T Moore A Dawany N. Ruxolitinib: targeted approach for treatment of autoinflammatory very early onset inflammatory bowel disease. Clin Gastroenterol Hepatol. (2021) 21:S1542–3565. 10.1016/j.cgh.2021.07.040 34329777 PMC8792097

[101] Connors J Basseri S Grant A Giffin N Mahdi G Noble A. Exclusive enteral nutrition therapy in paediatric crohn's disease results in long-term avoidance of corticosteroids: results of a propensity-score matched cohort analysis. J Crohns Colitis. (2017) 11:1063–70. 10.1093/ecco-jcc/jjx060 28575325 PMC5881686

[102] Diederen K Li JV Donachie GE de Meij TG de Waart DR Hakvoort T. Exclusive enteral nutrition mediates gut microbial and metabolic changes that are associated with remission in children with Crohn's disease. Sci Rep. (2020) 10:18879. 10.1038/s41598-020-75306-z 33144591 PMC7609694

[103] Hugot JP Chamaillard M Zouali H Lesage S Cezard JP Belaiche J. Association of NOD2 leucine-rich repeat variants with susceptibility to Crohn's disease. Nature. (2001) 411:599–63. 10.1038/35079107 11385576

[104] Kucuk ZY Bleesing JJ Marsh R Zhang K Davies S Filipovich AH. A challenging undertaking: stem cell transplantation for immune dysregulation, polyendocrinopathy, enteropathy, X-linked (IPEX) syndrome. J Allergy Clin Immunol. (2016) 137:953–5. 10.1016/j.jaci.2015.09.030 26559324

[105] Engelhardt KR Shah N Faizura-Yeop I Kocacik UD Frede N Muise AM. Clinical outcome in IL-10- and IL-10 receptor-deficient patients with or without hematopoietic stem cell transplantation. J Allergy Clin Immunol. (2013) 131:825–30. 10.1016/j.jaci.2012.09.025 23158016

[106] Kotlarz D Beier R Murugan D Diestelhorst J Jensen O Boztug K. Loss of interleukin-10 signaling and infantile inflammatory bowel disease: implications for diagnosis and therapy. Gastroenterol. (2012) 143:347–55. 10.1053/j.gastro.2012.04.045 22549091

[107] Murugan D Albert MH Langemeier J Bohne J Puchalka J Jarvinen PM. Very early onset inflammatory bowel disease associated with aberrant trafficking of IL-10R1 and cure by T cell replete haploidentical bone marrow transplantation. J Clin Immunol. (2014) 34:331–9. 10.1007/s10875-014-9992-8 24519095

[108] Tsuma Y Imamura T Ichise E Sakamoto K Ouchi K Osone S. Successful treatment of idiopathic colitis related to XIAP deficiency with allo-HSCT using reduced-intensity conditioning. Pediatr Transplant. (2015) 19:E25–8. 10.1111/petr.12405 25412586

[109] Lekbua A Ouahed J O'Connell AE Kahn SA Goldsmith JD Imamura T. Risk-factors associated with poor outcomes in VEO-IBD secondary to XIAP deficiency: A case report and literature review. J Pediatr Gastroenterol Nutr. (2019) 69:e13–8. 10.1097/MPG.0000000000002297 31232887 PMC6607918

[110] Chen R Giliani S Lanzi G Mias GI Lonardi S Dobbs K. Whole-exome sequencing identifies tetratricopeptide repeat domain 7A (TTC7A) mutations for combined immunodeficiency with intestinal atresias. J Allergy Clin Immunol. (2013) 132:656–64. 10.1016/j.jaci.2013.06.013 23830146 PMC3759618

[111] Klemann C Pannicke U Morris-Rosendahl DJ Vlantis K Rizzi M Uhlig H. Transplantation from a symptomatic carrier sister restores host defenses but does not prevent colitis in NEMO deficiency. Clin Immunol. (2016) 164:52–6. 10.1016/j.clim.2016.01.010 26812624 PMC6101191

[112] Lourenco R Azevedo S Lopes AI. Surgery in pediatric crohn disease: Case series from a single tertiary referral center. GE Port J Gastroenterol. (2016) 23:191–6. 10.1016/j.jpge.2016.03.007 28868459 PMC5580150

[113] Abraham BP Mehta S El-Serag HB. Natural history of pediatric-onset inflammatory bowel disease: a systematic review. J Clin Gastroenterol. (2012) 46:581–9. 10.1097/MCG.0b013e318247c32f 22772738 PMC3972042

[114] Pini-Prato A Faticato MG Barabino A Arrigo S Gandullia P Mazzola C. Minimally invasive surgery for paediatric inflammatory bowel disease: personal experience and literature review. World J Gastroenterol. (2015) 21:11312–20. 10.3748/wjg.v21.i40.11312 26525138 PMC4616207

[115] Paramsothy S Kamm MA Kaakoush NO Walsh AJ van den Bogaerde J Samuel D. Multidonor intensive faecal microbiota transplantation for active ulcerative colitis: A randomised placebo-controlled trial. Lancet. (2017) 389:1218–28. 10.1016/S0140-6736(17)30182-4 28214091

[116] Rossen NG Fuentes S van der Spek MJ Tijssen JG Hartman JH Duflou A. Findings from a randomized controlled trial of fecal transplantation for patients with ulcerative colitis. Gastroenterol. (2015) 110–8. 10.1053/j.gastro.2015.03.045 25836986

[117] Hourigan SK Oliva-Hemker M. Fecal microbiota transplantation in children: a brief review. Pediatr Res. (2016) 80: 2–6. 10.1038/pr.2016.48 26982451

