# Corrigendum: New Insights and Advances in Pathogenesis and Treatment of Very Early Onset Inflammatory Bowel Disease

**DOI:** 10.3389/fped.2022.894682

**Published:** 2022-05-12

**Authors:** Qi-Qi Li, Hui-Hong Zhang, Shi-Xue Dai

**Affiliations:** ^1^The Second School of Clinical Medicine, Southern Medical University, Guangzhou, China; ^2^Department of Gastroenterology, Guangdong Provincial Geriatrics Institute, National Key Clinical Specialty, Guangdong Provincial People's Hospital, Guangdong Academy of Medical Sciences, Guangzhou, China; ^3^Department of Gastroenterology, Guangdong Provincial People's Hospital, Guangdong Academy of Medical Sciences, South China University of Technology, Guangzhou, China

**Keywords:** microRNA, circular RNA, biologics, immunity, gut microbiota

In the original article, there was a mistake in Figure 1 as published. IL-2 and IFN-γ was originally described as a soluble factor released by CD4+ Th2. This should be changed to “IL-10 was described as a soluble factor released by CD4+ Th2 cells that can preclude the release of CD4+ Th1 cytokines, such as IL-2 and IFN-γ.” The corrected Figure 1 appears below.

**Figure 1:**
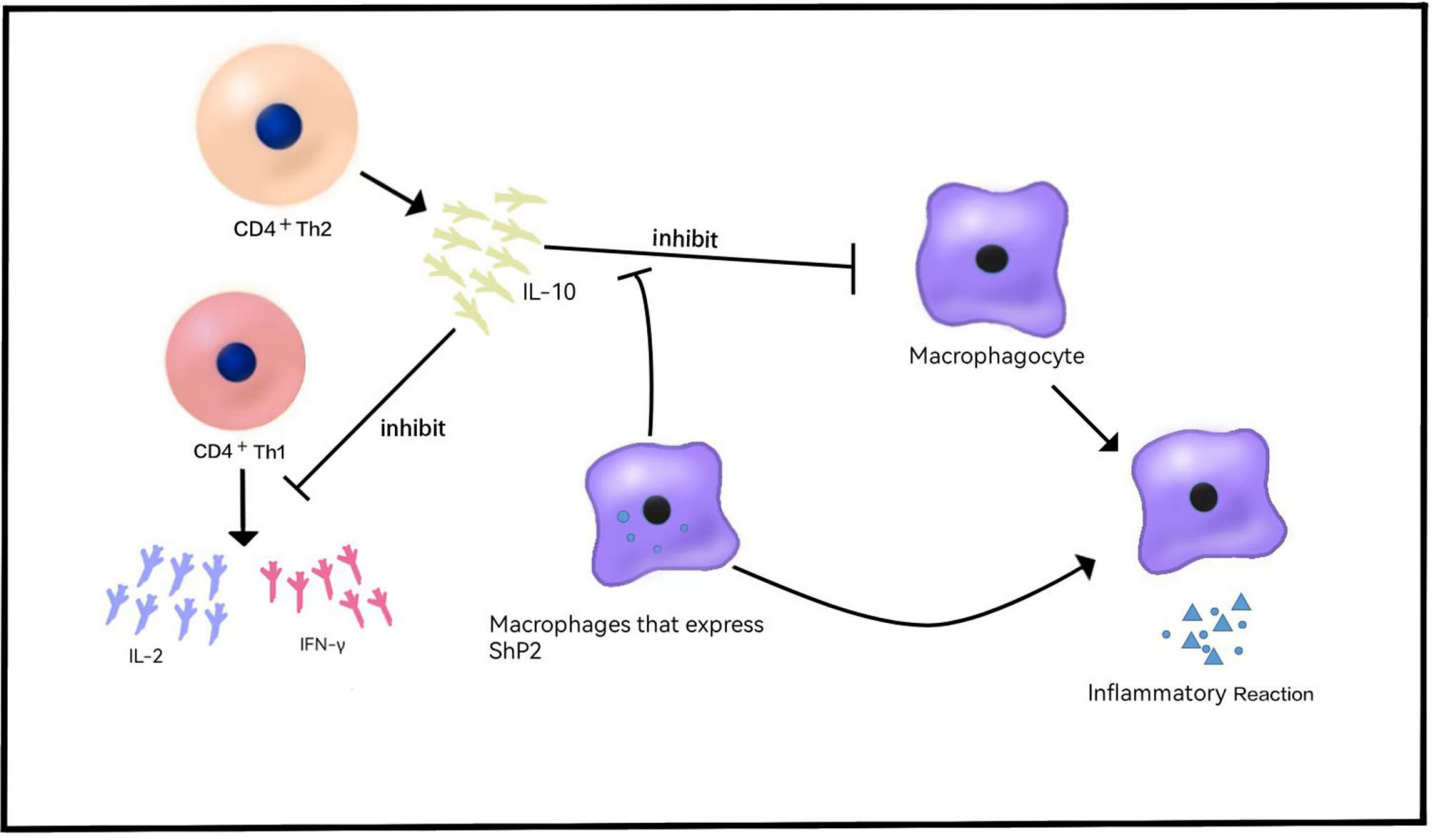
Role of IL-10 in VEO-IBD. IL-10 is released by CD4^+^Th2 cells and inhibits the release of cytokines such as IL-2 and IFN-γ. IL-10 inhibits the release of inflammatory cytokines and the inflammatory response. Shp2 can reduce the sensitivity of macrophages to IL-10 and produce proinflammatory effects.

In the original article, there was an error in **Immune Dysregulation**, “*Cytokines and Their Receptors*.” “IL-10 was originally described as a soluble factor released by CD4+ Th2 cells that can preclude the release of CD4+ Th4 cytokines, such as IL-2 and IFN-γ (22) (Figure 1)” has been corrected to “IL-10 was originally described as a soluble factor released by CD4+ Th2 cells that can preclude the release of CD4+ Th1 cytokines, such as IL-2 and IFN-γ (22) (Figure 1).”

The authors apologize for this error and state that this does not change the scientific conclusions of the article in any way. The original article has been updated.

## Publisher's Note

All claims expressed in this article are solely those of the authors and do not necessarily represent those of their affiliated organizations, or those of the publisher, the editors and the reviewers. Any product that may be evaluated in this article, or claim that may be made by its manufacturer, is not guaranteed or endorsed by the publisher.

## References

[22] Fiorentino DF Bond MW Mosmann TR. Two types of mouse T helper cell. IV. Th2 clones secrete a factor that inhibits cytokine production by Th1 clones. J Exp Med. (1989) 170: 2081–95. 10.1084/jem.170.6.2081 2531194 PMC2189521

